# Soft tissue aneurysmal bone cyst in thigh muscles: A case report

**DOI:** 10.1002/ccr3.6750

**Published:** 2022-12-15

**Authors:** Hossein Bozorgi, Behzad Aminzadeh, Amir Mahmoud Ahmadzade, Farzane Khoroushi, Mostafa Izanlu

**Affiliations:** ^1^ Department of Radiology, Faculty of Medicine Mashhad University of Medical Sciences Mashhad Iran; ^2^ Student Research Committee, Faculty of Medicine Mashhad University of Medical Sciences Mashhad Iran; ^3^ Department of Pathology Imam Reza Hospital, Mashhad University of Medical Sciences Mashhad Iran

**Keywords:** aneurysmal bone cyst, soft tissue aneurysmal bone cyst, soft tissue mass, thigh mass

## Abstract

Aneurysmal bone cysts (ABCs) are benign lesions that are locally invasive. The prevalence of soft tissue ABCs is far lower than the intraosseous type. Here, we report a case of ABC in thigh muscles who presented with distal thigh pain.

## Graphical abstract

A 16‐year‐old female presented with distal thigh pain. X‐ray revealed an oval‐shaped mass in the soft tissue of mid to distal thigh with calcified margins and internal cystic spaces. MRI images showed soft tissue mass containing multiple cystic areas and some fluid levels within the quadriceps femoris muscle. The lesion was resected en bloc. Multilocular cystic lesion filled with blood spaces and separated by fibrous septa interspersed with metaplastic woven bone with dystrophic calcification and osteoclastic giant cells without cytonuclear atypia was observed in histopathologic examination which favored the diagnosis of soft tissue aneurysmal bone cyst.

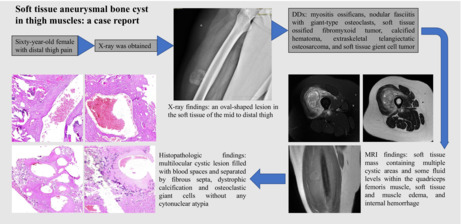

## 1 INTRODUCTION

For the first time, Jaffe and Lichtenstein described aneurysmal bone cyst (ABC) in 1942.^1^ ABCs are benign lesions that are locally invasive. They can also relapse locally. There is no preference in sex^2^ and race^1^ in the occurrence of this lesion. They can appear at any age, but in general, they are more common in the first two decades of life.^2^ The most expected locations for ABCs are the metaphysis of long bones and vertebral bodies.^3^ In MRI, they present as lytic lesions with sharp borders, bone expansion, and fluid–fluid level.^4^

Due to the association of these lesions with other benign and malignant pathologies, the initial idea was that reactive bone processes are the cause of their formation.^2^ On the other hand, many studies have published evidences in favor of the neoplastic nature of ABCs.^1^ Therefore, clonal processes have been suggested for primary aneurysmal cysts due to the discovery of translocation in the ubiquitin‐specific peptidase 6 (USP6) gene.^5^ t(16,17) (q22;p13) is the most common translocation found, which causes USP6 to be located near the osteoblastic cadherin 11 (CDH11) promoter.^6^

Probably the first description of soft tissue ABC was made by Salm and Sissons in 1972.^7^, ^8^ The prevalence of soft tissue ABC is far lower than the intraosseous type. These two types of ABCs have very similar histological features. Therefore, the diagnosis of soft tissue type depends on the clinical and imaging findings.^2^ This article is a report of a patient with soft tissue ABC with its clinical, imaging, and histological features, which helps physicians to differentiate this lesion and identify its features.

## 2 CASE PRESENTATION

The patient was a 16‐year‐old female who had pain in the distal thigh since 3 months before diagnosis. There was no history of special disease, previous surgery, and trauma. History of ABC in the patient's family was not reported.

After going to the physician and initial evaluations, the patient was given supportive treatment, but due to the lack of improvement, she was examined again by the physician after a few days and was recommended to do several physiotherapy sessions with the diagnosis of muscle cramps. Due to the lack of symptoms improvement after physiotherapy sessions and the feeling of a lump in the distal thigh, the patient was referred to a specialist physician, where she was asked for a thigh radiograph.

**FIGURE 1:**
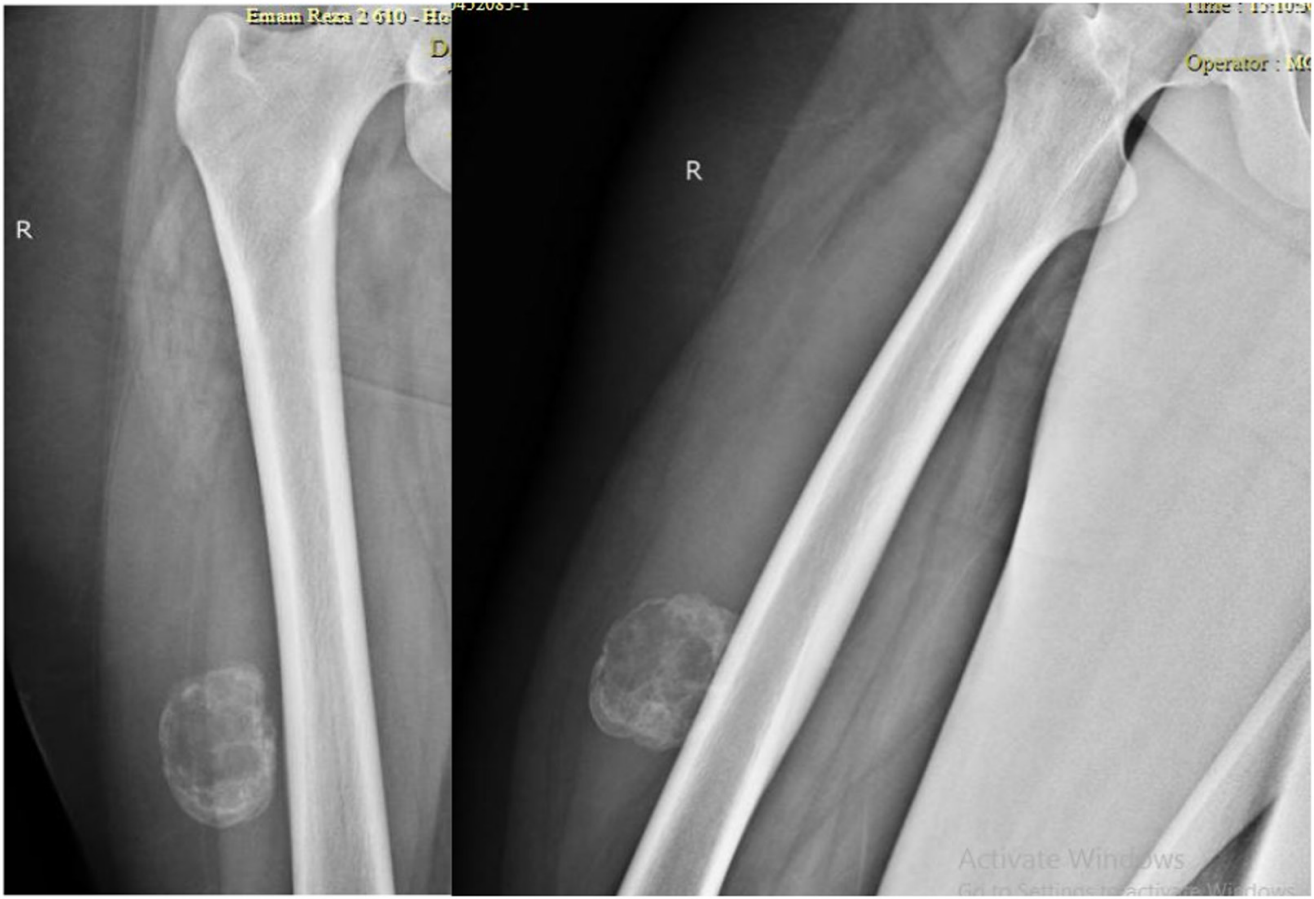
Right lower limb radiographs showing soft tissue mass with well‐defined calcified margins

**FIGURE 2:**
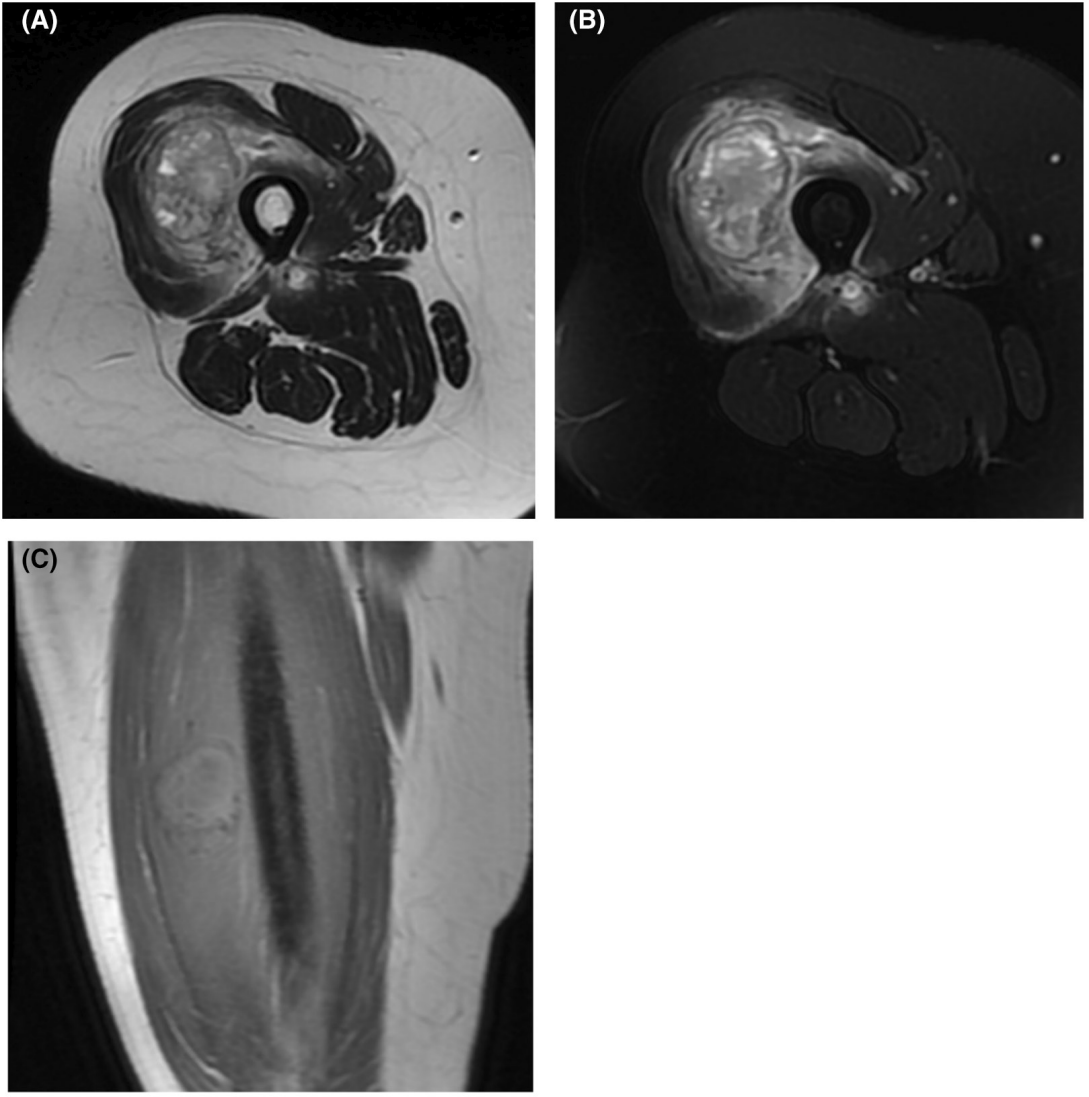
Axial T2W (A) and PD‐fat sat (B) images showing soft tissue mass with well‐defined hyposignal margins containing multiple cystic areas and some fluid levels within the quadriceps femoris muscle. Surrounding soft tissue and muscle edema is evident. (C) Coronal T1W image shows hypersignal internal content within the mass consistent with internal hemorrhage

In the x‐ray image, an oval‐shaped lesion measuring 4 cm (transvers) × 6 cm (craniocaudally) × 4.3 cm (anteroposterior) was seen in the soft tissue of the mid to distal thigh with well‐defined calcified margins and internal cystic spaces with no connection to the adjacent femoral bone (Figure 1). MRI images showed soft tissue mass with well‐defined hyposignal margins containing multiple cystic areas and some fluid levels within the quadriceps femoris muscle. Surrounding soft tissue and muscle edema was noted. T1 hypersignal internal content consistent with internal hemorrhage within the mass was evident (Figure 2). Bone scan showed soft tissue mass with increased tracer uptake at perfusion, blood pool, and delayed images.

Based on clinical evidence and imaging, the surgeon suspected benign soft tissue lesions. In the next step, the patient was a candidate for complete surgical resection of the lesion. The surgery was performed under general anesthesia. The lesion was resected en bloc after an anterolateral incision in the distal right thigh and passing through the vastus lateralis muscle. Finally, the muscles, skin, and subcutaneous tissue were repaired.

**FIGURE 3:**
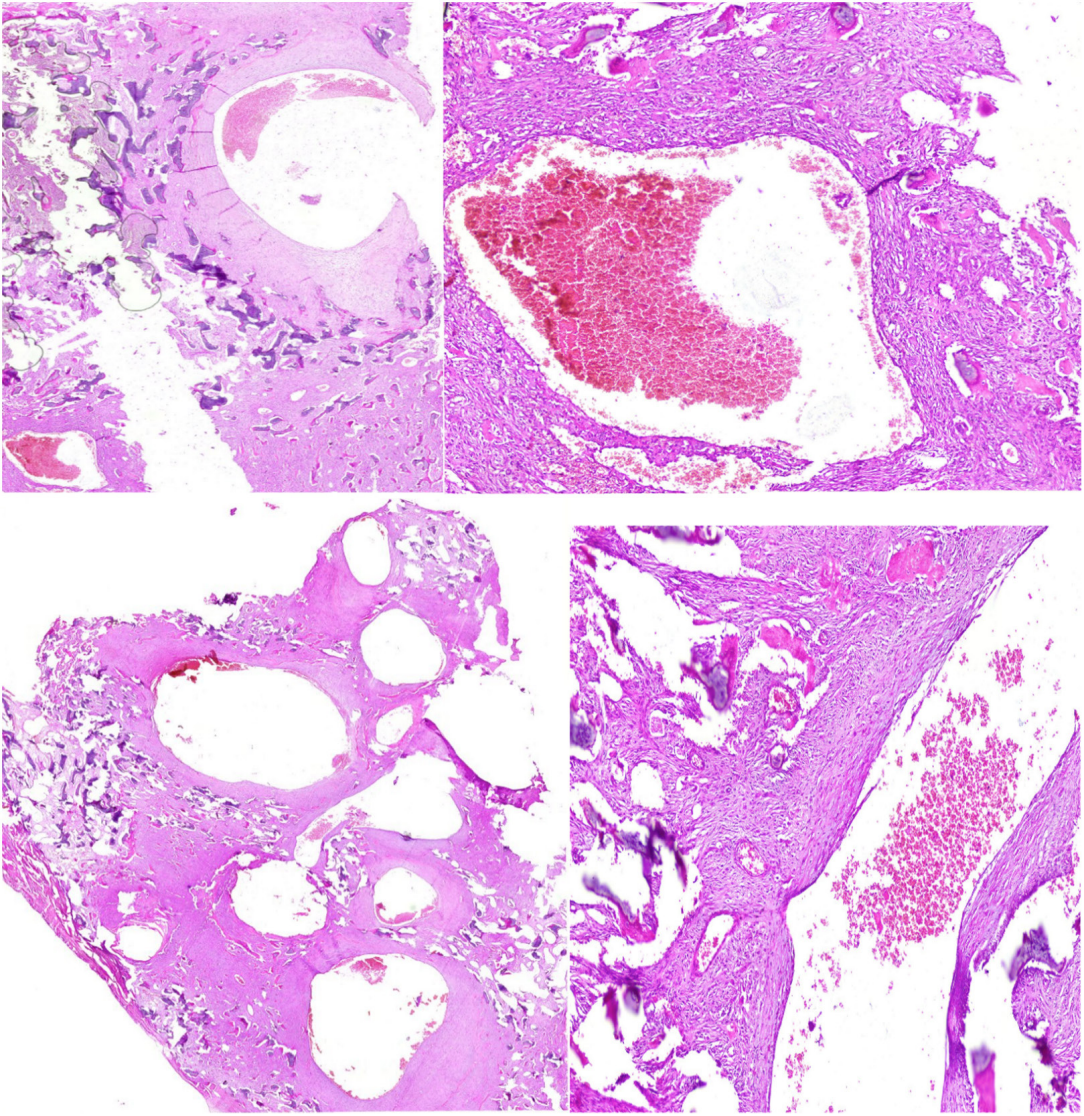
Multilocular cystic lesion separated by fibrous septa containing bland fibroblast/myofibroblast‐like spindle cells of severe cellularity. Some cysts are filled with blood

After resection of the lesion, a well‐circumscribed, tan‐white, multi‐cystic lesion filled with blood and variable solid components was seen in the gross evaluation. In microscopic examination of the resected sample stained with Hematoxylin and Eosin, multilocular cystic lesion filled with blood spaces and separated by fibrous septa containing bland fibroblast/myofibroblast‐like spindle cells of severe cellularity, interspersed with metaplastic woven bone with occasionally dystrophic calcification and osteoclastic giant cells without any cytonuclear atypia was observed which was suggestive of soft tissue ABC (Figure 3).

According to the radiological and clinical findings and the absence of any relationship between the lesion and the bone, the patient was diagnosed with soft tissue ABC. In the patient's follow‐up and the repeated clinical evaluations performed during 6 months, no evidence of recurrence was seen at the lesion site. Also, the patient did not mention any clinical complaints such as pain or swelling in the lesion site after resection. Moreover, in further evaluations, no evidence of recurrence in other parts of body or involvement of the bones was discovered.

## 3 DISCUSSION

The occurrence of aneurysmal bone cyst in soft tissue without any connection with bone has been previously mentioned in a number of published reports. It is very difficult to distinguish this lesion from other soft tissue lesions by clinical and radiological findings. Among soft tissue lesions that are clinically and radiologically in differential diagnosis with this lesion are myositis ossificans, nodular fasciitis with giant‐type osteoclasts, soft tissue ossified fibromyxoid tumor, calcified hematoma, extraskeletal telangiectatic osteosarcoma (EOS), and soft tissue giant cell tumor.^1^, ^9^

One of the most important differential diagnoses of soft tissue ABC is myositis ossificans, which have very similar radiological findings. In both, a radiolucent lesion with an ossified border is seen. The difference between them is the presence of septa in the internal cystic components in soft tissue ABC, while internal solid components are seen in myositis ossificans.^10^ Another important differential diagnosis is giant cell tumors. These tumors, especially when they undergo necrosis, hemorrhage, and cystic changes, have a radiological appearance similar to soft tissue ABC. Therefore, they can be distinguished from each other by histological findings.^11^ Another important tumor that may be confused with soft tissue ABC is EOS. The presence of an indistinct margins in radiological images is more in favor of EOS. Also, the presence of anaplastic cells and atypical mitosis helps in distinguishing this lesion from soft tissue ABC in histology.^10^

Typical sites of bony ABCs include spine (16%), metaphysis of bones like femur (13%), lower leg (24%), upper limbs (21%), and foot (3%). However, it can also occur in broad bones (4% skull and mandible, 12% hip and sacrum, 5% rib and clavicle).^12^ Of the 20 reported cases in the studies, 15 cases were found to have the lesion in the limbs proximal region (9 cases in the proximal upper limb and 6 cases in the proximal lower limb). Other cases were reported to have the lesion in the groin, abdominal wall, pelvis, and common carotid artery.^11^ In the presented case, the lesion was seen between the middle and distal third of the thigh.

In the report of Karkuzhali et al., multiple extraosseous aneurysmal cysts in the muscle and soft tissue of the right upper leg were reported in a 28‐year‐old man, which occurred 15 months after removal of the proximal fibula aneurysmal cyst.^9^ Therefore, a bony origin for soft tissue ABCs can be considered.

Aneurysmal bone cysts findings in MRI include cystic spaces filled with blood (causing fluid–fluid level appearance), low signal of septa in T1 and T2 (causing honeycombing appearance after contrast administration), and sharp low signal margin (due to calcification).^11^ The characteristic “donut sign” appears in nuclear scintigraphy of mature soft tissue ABC with calcified borders, which is caused by osteoblastic activity of the calcified borders and central photopenia of the central cystic areas.^13^

In the histological examination of soft tissue ABCs, multiple central cystic cavities separated by thin and thick septa containing fibroblasts, giant‐type osteoblasts, and bone spicules are seen which are very similar to the findings in intraosseous type of the lesion.^9^, ^11^ The margin of the lesion is ossified and can be seen in different stages of maturity.^9^

Clinical and imaging manifestations mimic many other soft tissue lesions. On the other hand, due to the low prevalence of soft tissue ABC, this lesion is less likely to be considered as a diagnosis. Paying attention to the location of these lesions, which often occur in the limbs, and considering it as a differential diagnosis of the lesions with myositis ossificans appearance can help in the diagnosis of this lesion.

## 4 CONCLUSION

The low prevalence of soft tissue ABC makes this lesion less likely to be considered. Due to the similarity of radiological and pathological appearance in soft tissue and intraosseous types, the combination of clinical, imaging, and pathological findings is necessary for the correct diagnosis of soft tissue ABC.

## AUTHOR CONTRIBUTIONS

**Hossein Bozorgi:** Conceptualization; data curation; methodology; writing – original draft. **Behzad Aminzadeh:** Conceptualization; data curation; methodology; writing – original draft. **Amir Mahmoud Ahmadzade:** Conceptualization; data curation; methodology; writing – original draft. **Farzaneh Khoroushi:** Conceptualization; data curation; methodology; writing – original draft. **Mostafa Izanlu:** Conceptualization; data curation; methodology; writing – review and editing.

## FUNDING INFORMATION

The authors did not receive any funding for this study.

## CONFLICT OF INTEREST

The authors declared no conflicts of interest.

## ETHICAL APPROVAL

This study was approved by the institutional review board committee.

## CONSENT

Written informed consent was obtained from the patient to publish this report in accordance with the journal's patient consent policy.

## Data Availability

The data that support the findings of this study are available from the corresponding author upon reasonable request.
